# Identification of thyroid tumor cell vulnerabilities through a siRNA-based functional screening

**DOI:** 10.18632/oncotarget.5282

**Published:** 2015-09-25

**Authors:** Maria Chiara Anania, Fabio Gasparri, Elena Cetti, Ivan Fraietta, Katia Todoerti, Claudia Miranda, Mara Mazzoni, Claudia Re, Riccardo Colombo, Giorgio Ukmar, Stefano Camisasca, Sonia Pagliardini, Marco A. Pierotti, Antonino Neri, Arturo Galvani, Angela Greco

**Affiliations:** ^1^ Molecular Mechanisms Unit, Fondazione IRCCS Istituto Nazionale dei Tumori, Milan, Italy; ^2^ Cell Biology Department, Nerviano Medical Sciences Srl, Nerviano (MI), Italy; ^3^ Laboratory of Pre-Clinical and Translational Research, IRCCS-CROB, Referral Cancer Center of Basilicata, Rionero in Vulture, Italy; ^4^ Scientific Directorate, Fondazione IRCCS Istituto Nazionale dei Tumori, Milan, Italy; ^5^ Department of Clinical Sciences and Community Health, University of Milan, Milan, Italy; ^6^ Hematology Unit, Fondazione IRCCS Ca’ Granda, Ospedale Maggiore Policlinico, Milan, Italy

**Keywords:** thyroid cancer, non-oncogene addiction, *MASTL*, *Cyclin D1*, *COPZ1*

## Abstract

The incidence of thyroid carcinoma is rapidly increasing. Although generally associated with good prognosis, a fraction of thyroid tumors are not cured by standard therapy and progress to aggressive forms for which no effective treatments are currently available. In order to identify novel therapeutic targets for thyroid carcinoma, we focused on the discovery of genes essential for sustaining the oncogenic phenotype of thyroid tumor cells, but not required to the same degree for the viability of normal cells (non-oncogene addiction paradigm). We screened a siRNA oligonucleotide library targeting the human druggable genome in thyroid cancer BCPAP cell line in comparison with immortalized normal human thyrocytes (Nthy-ori 3–1). We identified a panel of hit genes whose silencing interferes with the growth of tumor cells, while sparing that of normal ones. Further analysis of three selected hit genes, namely *Cyclin D1*, *MASTL* and *COPZ1*, showed that they represent common vulnerabilities for thyroid tumor cells, as their inhibition reduced the viability of several thyroid tumor cell lines, regardless the histotype or oncogenic lesion. This work identified non-oncogenes essential for sustaining the phenotype of thyroid tumor cells, but not of normal cells, thus suggesting that they might represent promising targets for new therapeutic strategies.

## INTRODUCTION

The incidence of thyroid cancer (TC), the most common endocrine malignancy, has increased significantly over the last few decades [1]. All TCs, except medullary thyroid carcinoma, are derived from thyroid follicular cells. Differentiated tumors, namely papillary and follicular carcinoma (PTC and FTC, 80% and 10% of cases, respectively) are generally cured by current treatment involving surgery, thyroid hormone and radioactive iodine therapy; however, they occasionally progress into undifferentiated, more aggressive and lethal carcinomas. Poorly differentiated (PDTC) and anaplastic (ATC) carcinoma, although rare, are the most clinically aggressive TCs; for ATC patient the survival is reduced to few months [2].

Genetic studies performed in the last 25 years have dissected the molecular bases of TC. PTC is associated with a high frequency of deregulation of RTK/RAS/RAF/MAPK pathway [3]. This includes oncogenic rearrangements of the *RET* and *NTRK1* receptors, and point mutations in the *BRAF* and *RAS* genes, with the *BRAFV600E* as the most frequent PTC alteration. The genetic landscape of PTC has been very recently expanded by integrated genomic characterization studies which identified several novel driver alterations [4]. FTC is associated with *RAS* mutations and *PAX8/PPARγ* rearrangements. *RAS* mutations are common in PDTC. ATC is associated with mutations of *BRAF*, *RAS*, *TP53*, *PTEN*, and *PIK3CA*. The identification and functional characterization of differentially expressed genes and miRNAs have also contributed to the understanding of thyroid tumor biology, and provided important diagnostic/prognostic tools [5–8].

Molecular findings have been translated into the clinical setting, leading to clinical experimentation of agents targeting thyroid oncoproteins or downstream pathways [9]. Several kinase inhibitors, primarily targeting BRAF and angiogenic kinases, are under evaluation in clinical trials [10]. Nevertheless, no effective targeted therapies are currently available for aggressive TC that are not curable by the standard therapeutic approaches.

The traditional approach for cancer target discovery is based on the identification of driver oncogenes through the characterization of the molecular alterations of primary tumors. However, according to the concept of “non-oncogene addiction” (NOA), the uncontrolled growth of cancer cells often relies on normal genes, that are not themselves oncogenes or otherwise mutated, whose activity is essential for tumor cells but not required to the same extent by normal cells [11]. NOA genes cannot be identified by genetic approaches aimed at identifying gene mutation/aberrancy, but rather by large-scale siRNA-based functional screening, a strategy which is nowadays widely used for the identification of tumor cell vulnerabilities to be explored for therapeutic purposes [12–15].

In order to identify novel, unforeseen molecular targets for thyroid carcinoma, in this study we have undertaken the screening of a siRNA oligonucleotide library in tumor (BCPAP) and normal (Nthy-ori 3–1) thyroid cell lines, and we have identified a set of genes whose silencing reduces the growth of tumor cells, while sparing growth of non-transformed cells of the same tissue origin. Among the top ranked genes, we selected *Cyclin D1 (CCND1)*, *MASTL* and *COPZ1*. All of them passed a secondary confirmation study, which unequivocally demonstrated the dependency of BCPAP cells upon their activity. Interestingly, we found that silencing of *Cyclin D1*, *MASTL* and *COPZ1* inhibits the growth of several further thyroid tumor cell lines.

## RESULTS

### Druggable genome siRNA screening

**Figure 1:**
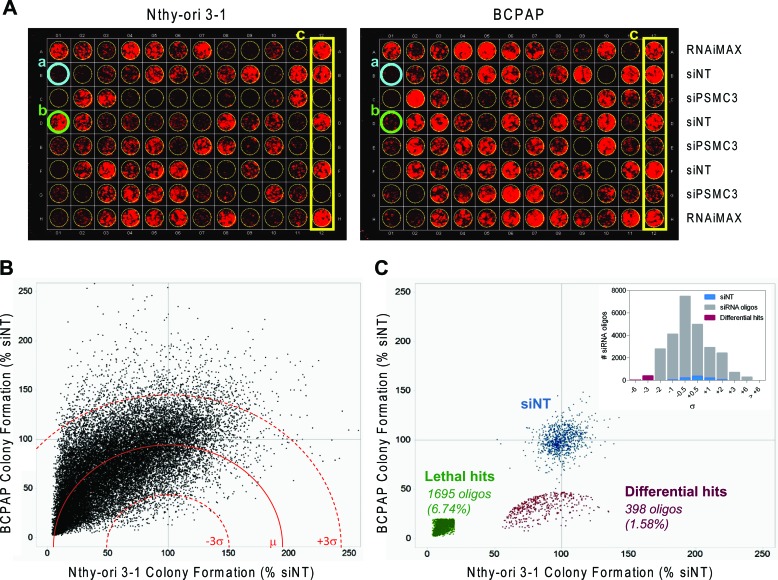
siRNA screening results **A.** Representative colony plates generated by transfecting Nthy-ori 3–1 (left) and BCPAP (right) cell lines with the same siRNA oligo mother plate. a. siRNA oligo lethal for both cell lines (blue); b. siRNA oligo selectively lethal for BCPAP (green); c. controls (yellow). **B.** Scatter plot of Colony Formation (CF) values obtained from 25139 unique siRNA oligos transfected in Nthy-ori 3–1 and BCPAP, reported as % of growth respect to NT for inter-plate data normalization (dot are the averages of two technical replicates performed in both cell lines). The mean *d* value (μ) is represented by a red line (dashed red lines: +/− 3σ). **C.** Scatter plot of normalized CF values showing the negative controls (siNT, blue), the lethal hits (green) and the differential hits (red). Insert: distribution of normalized *d* values after sigma binning.

To identify genes affecting growth of thyroid tumor cells, we conducted an RNAi-based phenotypic screening, examining effects on cell growth. The papillary thyroid carcinoma BCPAP cell line, carrying the *BRAFV600E* mutation, and the immortalized normal human thyrocyte Nthy-ori 3–1 cell line were transfected with a siRNA library containing 25139 siRNA oligos targeting about 9000 potentially “druggable” genes (3 duplexes/gene, on average), and with a non-targeting siRNA (siNT) and a siRNA targeting the proteasomal subunit *PSMC3* as negative and positive controls, respectively. Cells were transfected at low density in 96-well plates and colony formation assay (CFA) was performed after 7 (Nthy-ori 3–1) or 8 (BCPAP) days. Images of a representative plate for each of these lines are shown in Figure 1A. We preferred CFA to short-term (48–72 hours) proliferation assay, since it allows the detection of long-term consequences of “weak” phenotypes (our unpublished results). The screening results are shown in Figure 1B: scatter plots represent the fluorescence signal, derived from CFA acquisition, normalized with respect to siNT (% siNT) of Nthy-ori 3–1 and BCPAP cells transfected in duplicate with the library siRNA oligos (the complete list is reported in Table S1). Of note, the uneven distribution of data across graph diagonal denotes slightly higher transfection efficiency for Nthy-ori 3–1 than for BCPAP. Genes essential for cell viability of BCPAP, but not Nthy-ori 3–1 cells, were identified through the “*d* parameter” (defined in Materials and Methods). *d* values close to 0 denote preferential inhibition of BCPAP cell proliferation with respect to Nthy-ori 3–1. Based on data distribution, a threshold of −3σ (corresponding to *d* = 47.2) was applied to define differentially active hits: 398 siRNA oligos (1.58%), targeting 386 genes, were found to be below this threshold and thus were defined as “differential hits” (Figure 1C; hit list is reported in Table S2). A significant preferential activity towards BCPAP cells was observed for 12 genes with 2 oligos out of 3, and for the remaining 374 genes with 1 oligo out of 3; the latter include BRAF, consistent with the notion that BCPAP cells are addicted to *BRAFV600E* oncogene [16]. No genes emerged with 3/3 oligos among hits. Functional annotation clustering analysis was performed on the 386 gene list (382 DAVID IDs), using Gene Ontology-Biological Process (GO-BP) and Gene Ontology–Molecular Function (GO-MF) annotation terms and medium classification stringency. A significant Enrichment score (>1.3) was found in 15 out of the 117 annotation clusters that were globally identified. The top ranked GO-terms, representative for the 15 significant clusters, have been reported in Figure S1A.

By setting an arbitrary threshold of 20% colony growth with respect to siNT controls, we identified 1695 siRNA oligonucleotides (6.74%) capable of inhibiting cell growth both in BCPAP and Nthy-ori 3–1, therefore defined as “lethal hits” (Figure 1C). Two hundred and seventeen genes emerged as indiscriminately lethal hits with 2/3 (163) or 3/3 (54) oligonucleotides (Table S3). Most of them encode proteins involved in fundamental processes, and some, such as the kinases PLK1, WEE1, AURKB and several proteasome subunits, have previously been shown to be essential for cell survival, emerging as top-ranking lethal hits in RNAi-mediated phenotypic proliferation screens in different tumor cell lines [14, 17, 18].

### Confirmation of differentially active hits

**Figure 2:**
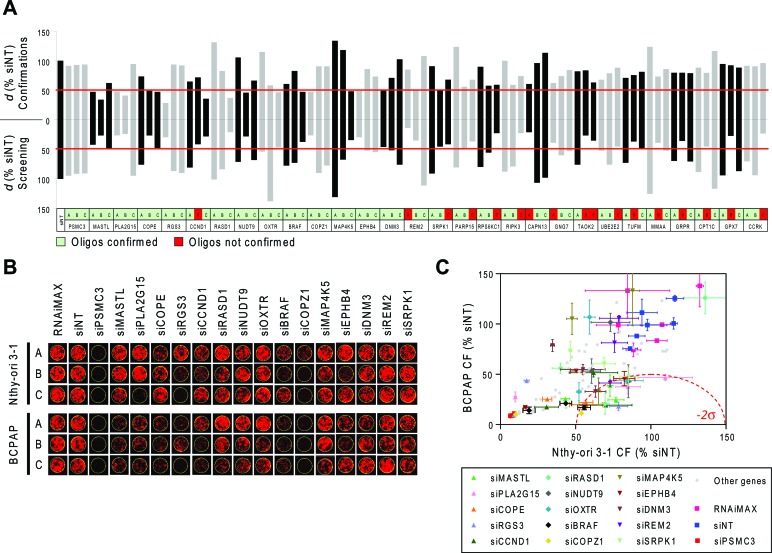
Confirmation of differentially active hits **A.** Comparison between *d* values obtained from the screening (bottom) and from confirmation experiments (top) performed with 84 siRNA oligos targeting 28 genes selected among the top ranked hits (A, B and C: three oligos per gene). Non-targeting Oligo (siNT) and siRNA oligos against PSMC3 were respectively used as negative and positive controls of transfection. Values represent the means of two technical replicates. The thresholds used to define differentially active oligos are shown in red (*d* = 47.2 for screening and *d* = 49.8 for confirmations). **B.** Representative pictures of individual wells showing colony formation in confirmation experiments after transfection of Nthy-ori 3–1 and BCPAP cells with the indicated siRNA oligos. **C.** Colony Formation (% siNT) values from image acquisition in B. All 84 oligos used for confirmation are shown; among these, oligos corresponding to the 15 genes are reported in B and are highlighted in color with standard deviation bars.

Eightyfour siRNA oligonucleotides, targeting 28 genes, were selected for confirmatory studies. Hits were prioritized for technical confirmation based on *d* values of individual oligos and on specific interest of identifying candidate druggable target genes. siRNAs were picked from library plates and transfected manually in triplicate on Nthy-ori 3–1 and BCPAP cells using the same experimental conditions as employed in primary screening. In these experiments a threshold of −2σ (corresponding to *d* = 49.8) was applied to define oligos with significantly low *d* values, i.e. displaying differential activity. We considered equally confirmed any siRNA oligos that in confirmation experiments gave the same phenotype of the screening, including the ones inactive or lethal for both cell lines. Figure 2A shows the comparison between primary screening and confirmation results. Eighteen out of 35 siRNA oligos (targeting 15 genes), which had shown differential activity (*d* < −3σ) in the primary screening were confirmed by manual transfection (*d* < −2σ). All of 49 oligos which were not found to be differentially active in the screening run (*d* > −3σ) were confirmed as such by manual transfections (*d* > −2σ). Overall, the phenotype of 67 out of 84 oligos was confirmed. Representative images of outgrown colonies from the confirmatory study are shown in Figure 2B and the corresponding quantifications are reported in Figure 2C. The list of confirmed hits is shown in Table 1: 3 genes were confirmed with 2 oligos (*MASTL*, *PLA2G15* and *COPE*) and 12 genes were confirmed with 1 oligo (*RGS3*, *CCND1*, *RASD1*, *NUDT9*, *OXTR*, *BRAF*, *COPZ1*, *MAP4K5*, *EPHB4*, *DNM3*, *REM2*, *SRPK1*). Functional annotation analysis indicates that these genes are involved in several biological processes, such as cell cycle control, DNA damage and cell death, vesicular transport and endocytosis, cellular metabolic processes and intracellular signal transduction (Table 1). Network analysis performed by Ingenuity Pathway Analysis (IPA) identified two major connection networks as reported in Figure S1B.

**Table 1:** List of the confirmed hits after the primary screening

Gene Symbol	Full Gene Name	Accession	GO-BP Term	Confirmed active oligos	*d* (active oligos)
***MASTL***	microtubule associated serine/threonine kinase-like	NM_032844	**GO:0006468** protein amino acid phosphorylation **GO:0006793** phosphorus metabolic process	2/2	33.75 (B) - 47.36 (A)
***PLA2G15***	phospholipase A2, group XV	NM_012320	**GO:0006575** cellular amino acid derivative metabolic process **GO:0006643** membrane lipid metabolic process	2/2	40.56 (B) - 47.71 (A)
***COPE***	coatomer protein complex, subunit epsilon	NM_007263	**GO:0006890** retrograde vesicle-mediated transport, Golgi to ER transport **GO:0051640** organelle localization	2/2	47.40 (C) - 48.45 (B)
***RGS3***	regulator of G-protein signaling 3	NM_017790	**GO:0000165** MAPKKK cascade **GO:0008277** regulation of G-protein coupled receptor proteinsignaling pathway	1/1	28.04 (A)
***CCND1***	Cyclin D1	NM_053056	**GO:0000075** cell cycle checkpoint **GO:0000077** DNA damage checkpoint	1/2	35.57 (C)
***RASD1***	RAS, dexamethasone-induced 1	NM_016084	**GO:0007264** small GTPase mediated signal transduction **GO:0016481** negative regulation of transcription	1/1	36.74 (C)
***NUDT9***	nudix (nucleoside diphosphate linked moiety X)-type motif 9	NM_024047	**GO:0006163** purine nucleotide metabolic process **GO:0006811** ion transport	1/1	45.6 (B)
***OXTR***	oxytocin receptor	NM_000916	**GO:0000165** MAPKKK cascade **GO:0003012** muscle system process	1/1	46.59 (C)
***BRAF***	v-raf murine sarcoma viral oncogene homolog B1	NM_004333	**GO:0000165** MAPKKK cascade **GO:0010941** regulation of cell death	1/1	47.45 (C)
***COPZ1***	coatomer protein complex, subunit zeta 1	NM_016057	**GO:0006886** intracellular protein transport **GO:0006890** retrograde vesicle-mediated transport, Golgi to ER	1/1	47.46 (A)
***MAP4K5***	mitogen-activated protein kinase kinase kinase kinase 5	NM_006575	**GO:0000165** MAPKKK cascade **GO:0001932** regulation of protein amino acid phosphorylation	1/1	47.88 (C)
***EPHB4***	EPH receptor B4	NM_004444	**GO:0007169** transmembrane receptor protein tyrosine kinase signaling pathway **GO:0045765** regulation of angiogenesis	1/1	48.98 (A)
***DNM3***	dynamin 3	NM_015569	**GO:0006897** endocytosis **GO:0010324** membrane invagination	1/1	49.29 (A)
***REM2***	RAS (RAD and GEM)-like GTP binding 2	NM_173527	**GO:0006355** regulation of transcription, DNA-dependent **GO:0007264** small GTPase mediated signal transduction	1/2	49.33 (B)
***SRPK1***	SFRS protein kinase 1	NM_003137	**GO:0006396** RNA processing **GO:0007243** protein kinase cascade	1/2	49.80 (B)
***PARP15***	poly (ADP-ribose) polymerase family, member 15	NM_152615		0/1	–
***RPS6KC1***	ribosomal protein S6 kinase, 52kDa, polypeptide 1	NM_012424		0/1	–
***RIPK3***	receptor-interacting serine-threonine kinase 3	NM_006871		0/1	–
***CAPN13***	calpain 13	NM_144575		0/1	–
***GNG7***	guanine nucleotide binding protein (G protein), gamma 7	NM_052847		0/1	–
***TAOK2***	TAO kinase 2	NM_004783		0/2	–
***UBE2E2***	ubiquitin-conjugating enzyme E2E 2 (UBC4/5 homolog, yeast)	NM_152653		0/1	–
***TUFM***	Tu translation elongation factor, mitochondrial	NM_003321		0/1	–
***MMAA***	methylmalonic aciduria (cobalamin deficiency) cblA type	NM_172250		0/1	–
***GRPR***	gastrin-releasing peptide receptor	NM_005314		0/1	–
***CPT1C***	carnitine palmitoyltransferase 1C	NM_152359		0/1	–
***GPX7***	glutathione peroxidase 7	NM_015696		0/1	–
***CCRK***	cell cycle related kinase	NM_012119		0/1	–

Genes are listed according to the number of active oligos; A, B and C represent three different oligos targeting the same gene. *d* values (<49.8) for each oligo, from the confirmation experiment, are also reported. NM: RefSeq Accession Number (http://www.ncbi.nlm.nih.gov/refseq/about/). Representative Gene Ontology-Biological Process (GO-BP) terms are selected for each of the 15 validated genes, by means of DAVID6.7 functional annotation analysis.

### Molecular profiling of confirmed hit genes

**Figure 3:**
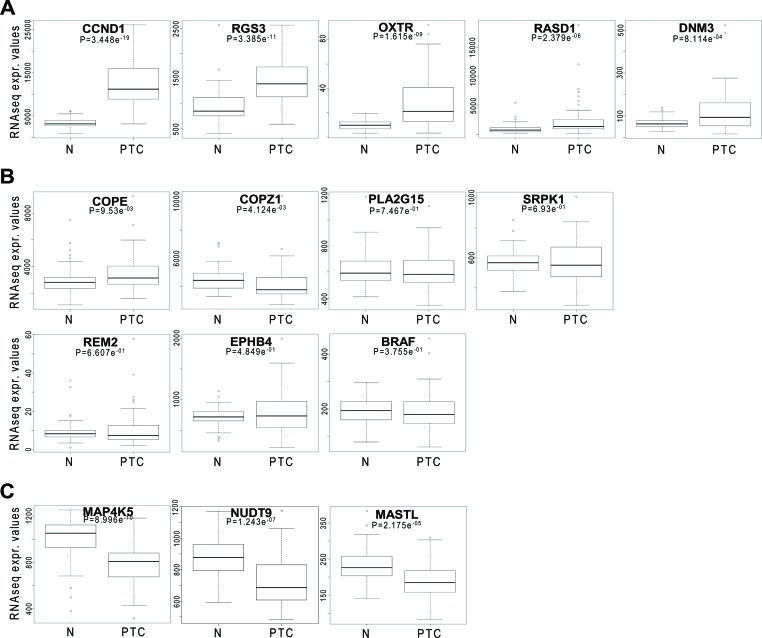
Hit gene expression levels in PTC Box plot distribution of RNAseq normalized expression values from the thyroid TCGA dataset of 15 selected genes in 58 matched normal (N) and PTC samples; **A, B** and **C.** represent genes overexpressed, equally or downregulated in PTC vs normal samples, respectively. The statistical significance of differences among the groups was assessed using Wilcoxon test.

The thyroid TCGA data set [4], including 496 PTC samples, 58 of which matched with normal thyroid, was interrogated for assessing the mutational status and the expression level of hit genes in PTC. As expected, oncogenic *BRAF* mutations were detected with high frequency, being present in 248 samples. No mutations affecting the other genes were detected, with the exception of a silent mutation in *COPZ1* gene in one case (data not shown). Expression analysis of hit genes was investigated on the whole data set (data not shown), as well as on the 58 matched tumor and normal samples (Figure 3); in both cases identical results were obtained. According to their expression levels, hit genes can be classified in three groups: 1) genes with significantly high overexpression in PTC vs normal thyroid: *CCND1*, *RGS3*, *OXTR*, *RASD1*, *DNM3*; 2) genes with equal or slightly different expression in PTC and normal thyroid: *COPE*, *COPZ1*, *PLA2G15*, *SRPK1*, *REM2*, *EPHB4*, *BRAF*; 3) genes that are significantly downregulated in PTC vs normal thyroid: *MAP4K5*, *NUDT9*, *MASTL*. No differences among different PTC variants (classical, follicular, tall cell) or tumor stage were observed (data not shown).

Data retrieved from the Cancer Cell Line Encyclopedia (http://www.broadinstitute.org/ccle/home) showed that all the hit genes are expressed in BCPAP cells, with levels ranging from “low” to “very high” (data not shown). Mutation analysis data indicated a low number (50/1651) of mutated genes in BCPAP cells. *Cyclin D1*, *MASTL*, *MAP4K5*, *SRPK1* and *EPHB4* genes were found to be of wild-type status, whereas no information was available for the remaining hit genes. These findings suggest that the addiction of BCPAP cells to the activity of the hit genes is unlikely related to alterations of the cognate proteins caused by mutations.

### Hit gene expression in thyroid cell lines

**Figure 4:**
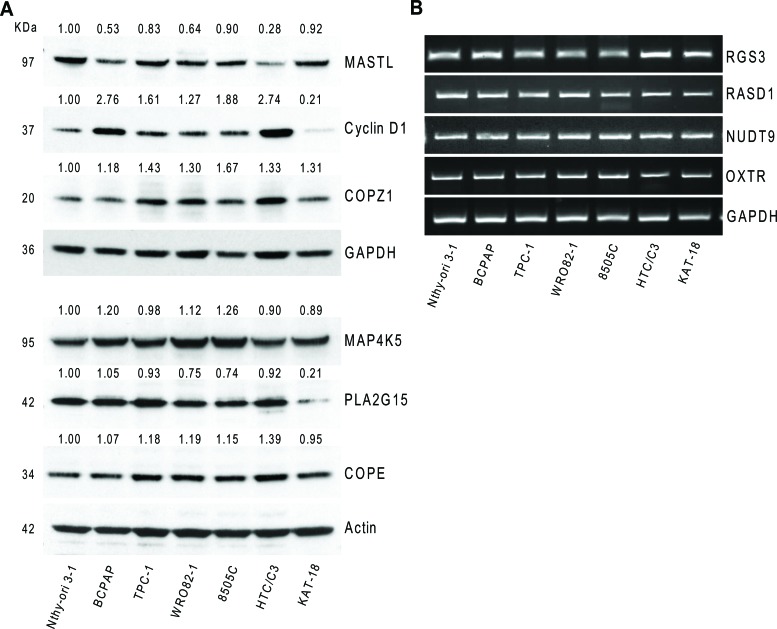
Hit gene expression in different thyroid tumor cells **A.** Western blot analysis of MASTL, Cyclin D1, COPZ1, MAP4K5, PLA2G15 and COPE protein expression in a panel of thyroid cell lines; values represent band intensity, assessed by densitometry analysis, reported as the ratio to actin (loading control) and normalized for the Nthy-ori 3–1 value. **B.** *RGS3*, *RASD1*, *NUDT9* and *OXTR* mRNA expression by RT-PCR in a panel of thyroid cell lines; *GAPDH* was used as housekeeping control. One out of three representative experiments is shown.

We investigated the expression of 10 genes (the top 11 hits except *BRAF*, see Table 1), in the normal (Nthy-ori 3–1) and tumor (BCPAP) cell lines used for the siRNA library screening, as well as in a panel of thyroid tumor cell lines, representative of different tumor histotypes, and/or carrying different oncogenes: PTC-derived (TPC-1, carrying the *RET/PTC1* oncogene); FTC-derived (WRO82–1); ATC-derived (8505C and HTC/C3, carrying the *BRAFV600E* oncogene; KAT-18). The expression of MASTL, PLA2G15, COPE, Cyclin D1, COPZ1 and MAP4K5 was investigated by western blot analysis (Figure 4A). Cyclin D1 was found to be 1.3 to 2.7 fold overexpressed in 6 out of 7 tumor cell lines with respect to Nthy-ori 3–1, with the exception of KAT-18, in which it was found less expressed. For COPE a slight increase in the protein expression levels in tumor cells, except KAT-18, with respect to Nthy-ori 3–1 was observed. MASTL was found frequently less expressed in tumor cells, in keeping with what was observed by gene expression analysis in tumor samples (Figure 3). COPZ1 protein expression was slightly increased in all the tumor cell lines, and greater in 8505C. PLA2G15 expression was slightly reduced in all the tumor cell lines, except BCPAP, and strongly reduced in KAT-18. For MAP4K5, a slight increase in BCPAP, WRO82–1 and 8505C, and a decrease in the remaining cell lines were observed.

The expression of *RASD1*, *NUDT9*, *RGS3* and *OXTR* genes was investigated by RT-PCR, as we failed to unequivocally detect the specific protein by western blot analysis (data not shown). As shown in Figure 4B, the genes are expressed in all the cell lines analyzed, and no differences can be appreciated.

### Hit validation in BCPAP cell line

**Figure 5:**
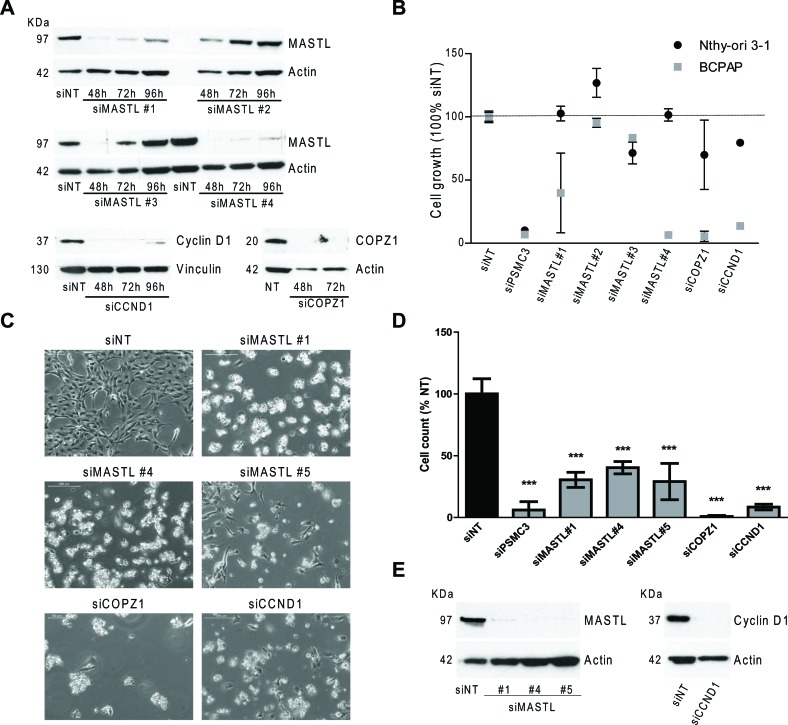
Effect of *MASTL*, *CCND1* and *COPZ1* silencing on BCPAP cells **A.** Western blot analysis of MASTL, Cyclin D1 and COPZ1 protein expression in Nthy-ori 3–1 cells, transiently transfected with siRNAs; actin was used as loading control. **B.** Scatter plot graph representing the percentage of cell growth (luminescence signal by CellTiter-Glo assay), normalized to that of Non Targeting oligo (siNT) 100%, 10 days after siRNA transfection in Nthy-ori 3–1 and BCPAP cells; siPSMC3 was used as positive control; one out of three representative experiments is shown. **C.** Representative pictures at 10X magnification of BCPAP cells 6 days after siRNA transfection. **D.** Cell proliferation assay of BCPAP cells 6 days after siRNAs transfection; the growth rate was determined by the trypan blue exclusion assay. Cell count of viable cells was normalized to that of siNT (100%); data represent the mean +/− sd of two independent experiments; the asterisks indicate differences significant by the unpaired Student's *t*-test (**P < 0.01, ***p < 0.001). **E.** Western blot analysis of MASTL and Cyclin D1 expression performed in the same samples of panel D; actin was used as loading control for cell extracts.

Among the candidate BCPAP vulnerability genes identified by the screening we selected *CCND1*, *MASTL* and *COPZ1* for a validation study using commercially available siRNAs, different from those of the library screening. Four different oligos for *MASTL*, and a smart pool of oligos for *CCND1* and *COPZ1* were used (details are reported in Table S4). Firstly, the efficiency of gene knock-down was evaluated by investigating the expression of corresponding proteins in Nthy-ori 3–1 cells transiently transfected with specific siRNAs and with non-targeting oligos (siNT) as control (Figure 5A). For MASTL the highest silencing efficiency was obtained with oligos 1 and 4 (89 and 95% of inhibition at 72 hours, respectively). siRNA targeting *COPZ1* and *CCND1* completely abrogated protein expression. Of note, in keeping with the siRNA library screening results, no macroscopic effects on Nthy-ori 3–1 cell growth/survival were observed (data not shown).

To investigate the effect of *MASTL*, *CCND1* and *COPZ1* gene silencing on BCPAP and Nthy-ori 3–1 cell viability, cells were transfected in 96 well plates with the different oligos above described, or with NT and PSMC3 siRNAs as controls, and analyzed by CellTiter-Glo assay 10 days later. Scatter plots in Figure 5B represent the luminescence signal (average of two replicates, normalized with respect to siNT) of cells transfected with each siRNA oligo. siMASTL#1, siMASTL#4, siCCND1 and siCOPZ1 reduced cell viability by 60%, 93%, 94% and 86%, respectively, an efficiency comparable to that of the control siRNA PMSC3 (93%). No effects were observed for siMASTL#2 and siMASTL#3, in keeping with their low silencing efficiency (Figure 5A). The effect of *MASTL*, *CCND1*, *COPZ1* gene silencing in BCPAP cells was further confirmed by a different experimental approach. Cells were transfected in 6-well plates with the oligos which showed efficient silencing in the previous experiment (siCCND1 and siCOPZ1 smart pools, siMASTL #1 and #4 oligos), and the newly in house synthesized MASTL oligo #5 (see Figure S2 for silencing efficiency). Six days later the effect of *MASTL*, *COPZ1* and *CCND1* silencing on both cell growth and cell viability was evident by microscope observation: silenced BCPAP cultures were less confluent than control (siNT); in addition, they presented numerous floating (most likely dead) cells (Figure 5C). Trypan blue exclusion assay showed a reduction of cell growth by 69.5%, 59.7% and 71% for siMASTL#1, #4 and #5, respectively; by 99.6% for siCOPZ1, by 91.5% for siCCND1 in comparison with the control (siNT). Western blot analysis performed at the end point of the experiment (Figure 5E) showed the complete abrogation of MASTL and Cyclin D1 protein expression. The same analysis was not feasible for COPZ1: due to the massive lethal effect of siCOPZ1, an insufficient amount of protein extract was recovered (data not shown).

Overall these results confirmed the growth inhibitory effect of *Cyclin D1*, *MASTL*, and *COPZ1* gene silencing, thus providing evidence that BCPAP cells are addicted to the activity of these genes.

### Effect of *Cyclin D1*, *MASTL* and *COPZ1* gene silencing on different thyroid tumor cell lines

Irrespective of the specific oncogenic driving lesion and histotypes, thyroid tumors may share common effector pathways for sustaining their transformed phenotype. To assess whether the survival genes confirmed in BCPAP cells are involved in such common pathways, we investigated the effect of *CCND1*, *MASTL* and *COPZ1* gene silencing in a panel of thyroid tumor-derived cell lines representative of different tumor histotypes.

**Figure 6:**
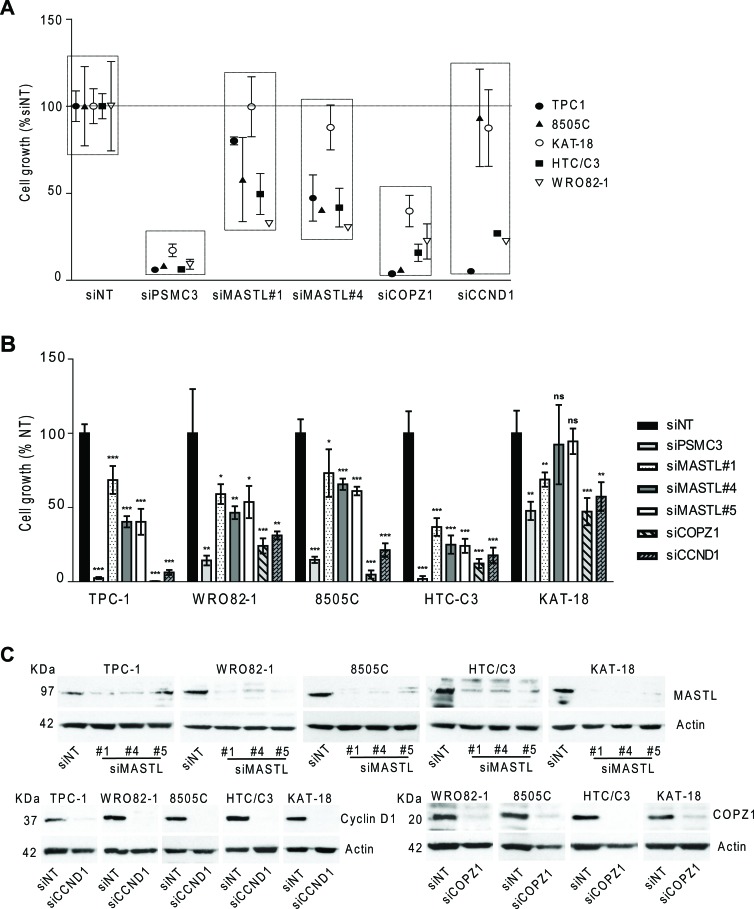
Effect of *CCND1*, *MASTL* and *COPZ1* silencing on different thyroid tumor cell lines **A.** Scatter plot graph representing the percentage of cell growth (luminescence signal by CellTiter-Glo assay), normalized to that of Non Targeting oligo (siNT) 100%, 10 days after siRNA transfection in a panel of thyroid cell lines; siPSMC3 was used as positive control. One out of three representative experiment is shown. **B.** Cell proliferation assay 4 days (for TPC1, 8505C and HTC/C3 cells), 5 days (for KAT-18 cells), 6 days (for BCPAP cells), 7 days (for WRO82–1 cells) after siRNAs transfection; the growth rate was determined by the trypan blue exclusion assay. Cell count of viable cells was normalized to that of siNT (100%); data represent the mean +/− sd of two independent experiments; the asterisks indicate differences significant by the unpaired Student's *t*-test (*P < 0.05, **P < 0.01, ***p < 0.001). **C.** Western blot analysis of MASTL, Cyclin D1 and COPZ1 expression performed in the same samples of panel B; actin was used as loading control for cell extracts.

Cell lines were transfected in 96 well plates with oligos targeting *MASTL*, *CCND1* and *COPZ1* genes and analyzed by CellTiter-Glo assay 10 days later. As reported in Figure 6A, gene silencing induced variable extents (15–95%) of cell growth inhibition in the majority of cell lines tested; no effect of *CCND1* silencing in 8505C and KAT-18 cells, and of *MASTL* silencing in KAT-18 was observed. We also assessed viability of cells transfected in 6-well plates by trypan blue exclusion assay performed four-seven days after siRNAs transfection. As reported in Figure 6B, in all the cell lines analyzed (except KAT-18), *MASTL*, *COPZ1* and *CCND1* gene silencing led to a significant decrease of cell viability: by 30–63% for siMASTL#1, by 35–75% for siMASTL#4, by 40–75% for siMASTL#5, by 75–99% for COPZ1 and by 70–94% for siCCND1. KAT-18 cells were found to be less sensitive to all the siRNA oligos: we found a decrease of cell growth by 31% for siMASTL#1, by 53% for si siCOPZ1 and by 43% for CCND1, and by 52.3% for the siPSMC3 control, and no effect for siMASTL#4 and #5. The reduction of cell growth in all the cell lines was also documented by images taken before cell count: silenced cells appeared less confluent in comparison with the control, and presented variable fractions of detached and floating cells (Figure S3). Concomitantly, western blot in Figure 6C shows the high efficiency of *MASTL*, *CCND1* and *COPZ1* silencing in the same samples. The same analysis was not feasible for TPC1 cells transfected with siCOPZ1, due to massive lethal effect (data not shown). Importantly, this experiment revealed a growth inhibitory effect of *CCND1* silencing in 8505C and KAT18, and of *MASTL* silencing in KAT18 cells, which was not previously observed. This could be ascribed to variability in the experimental conditions and/or silencing efficiency.

We next performed some experiments to further corroborate the dependency of thyroid cancer cells upon Cyclin D1, MASTL and COPZ1 activity.

**Figure 7:**
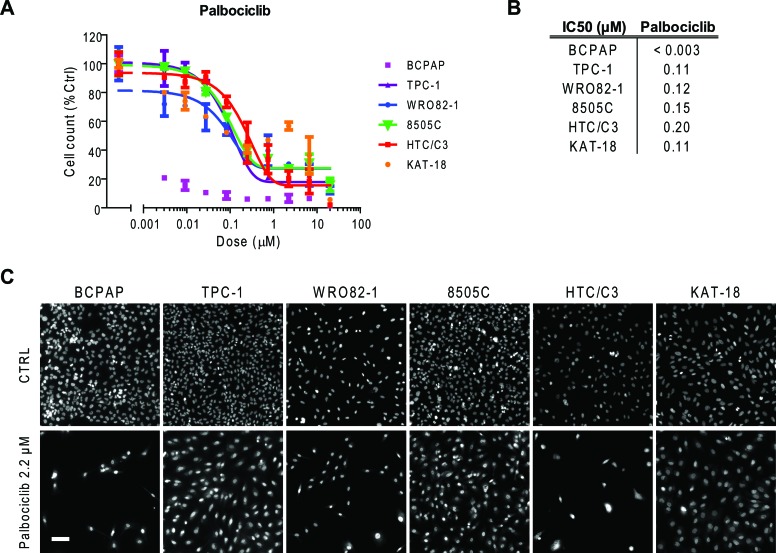
Thyroid cancer cell line sensitivity to palbociclib **A.** Proliferation dose response curves of thyroid cancer cell lines treated with palbociclib for 72 hours. Nuclei were stained with Hoechst and counted in each well by means of an ArrayScan high-content screening reader. The cell count per field, derived from nuclear staining, was reported as proliferative parameter and normalized with respect to untreated controls (100%). Each value represents the mean and standard deviation of two replicates. **B.** IC50 values (μM) obtained using a sigmoid function for nonlinear interpolation of experimental points; for KAT-18, the IC50 value was derived from graphical analysis. **C.** Representative ArrayScan fields of the thyroid cancer cell lines untreated (CTRL) or treated with 2.2 μM palbociclib for 72 h and stained with Hoechst (bar = 50 μm).

We tested thyroid tumor cell lines for susceptibility to palbociclib (PD-0332991), a specific inhibitor of Cyclin D1 associated kinases CDK4/6 [19]. Treatment with increasing doses of palbociclib showed a consistent antiproliferative effect in thyroid cancer cells, with an IC_50_ value ranging from <0.003 μM for BCPAP cells to 0.20 μM for HTC/C3 cells (Figure 7A and 7B). Representative images of thyroid cancer cell lines untreated or treated with 2.2 μM palbociclib for 72 h are reported in Figure 7C.

**Figure 8:**
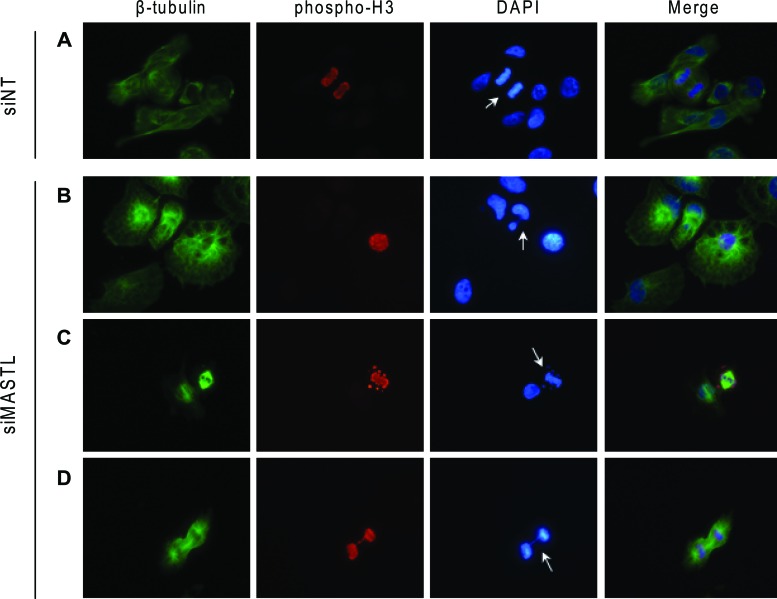
*MASTL* silencing causes abnormal mitotic figures 8505C cells transfected with the indicated siRNAs were analyzed by immunofluorescence for β-tubulin (green), phospho-histone H3 (red) and DAPI (DNA, blue). Arrows indicate normal mitosis **(A),** multinuclear cell **(B),** condensed chromosomes not correctly congressed to metaphase plate **(C)** DNA bridge **(D)** (Magnification 40x).

It has been reported that inhibition of *MASTL* induces multiple mitotic defects [20]. To investigate this issue in MASTL-depleted thyroid tumor cells, we performed the analysis reported in Figure 8. Asynchronous 8505C cells were transfected with the siMASTL or NT siRNA oligos and, 48 hours later, analyzed by immunofluorescence for β-tubulin, phospho-histone H3, and DNA for the presence of mitosis and nuclei aberrations. Several abnormalities associated with *MASTL* knockdown [20] were observed in MASTL-depleted 8505C cells. These include: multinuclear cells (panel B); abnormal mitotic figures, documented by the presence of condensed chromosomes that did not correctly align to metaphase plate (panel C); and chromatin bridges connecting daughter cells during anaphase (panel D). None of these abnormalities was observed in the control siNT cells (panel A). These results suggest that the growth inhibitory effect of *MASTL* silencing in thyroid tumor cells may be the consequence of failure of correct cell division process.

**Figure 9:**
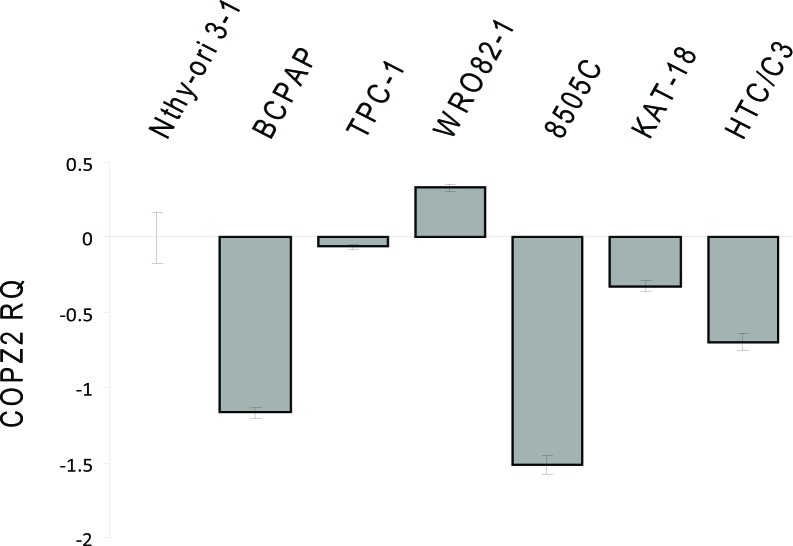
Expression of *COPZ2* in thyroid tumor cell lines Real-time PCR analysis of COPZ2 gene expression; results are presented as log10-transformed relative quantity (RQ) of COPZ2 mRNA normalized for HPRT1 housekeeping gene expression. Data represent the mean +/− sd of three independent experiments.

Shtutman et al. [21] have recently reported that tumor cell *COPZ1* vulnerability is related to downregulation of the paralogous *COPZ2* gene. To assess whether this occurs also in thyroid tumor cells we investigated the expression of *COPZ2* by Real Time PCR (Figure 9). With respect to control Nthy-ori 3–1 all the tumor cell lines analyzed, but WRO 82–1, showed downregulation of *COPZ2* mRNA. This result indicates that for the majority of thyroid tumor cell lines the *COPZ1* dependence may result from downregulation of *COPZ2*.

Collectively, our study identified Cyclin D1, MASTL and COPZ1 as thyroid cancer cell specific vulnerabilities, suggesting that they could provide a common therapeutic target in thyroid cancer treatment.

## DISCUSSION

Despite much progress having been made in the genetic and molecular characterization of thyroid carcinoma, few or no therapeutic options are currently available for patients with aggressive and iodine-refractory thyroid tumors. Kinase inhibitors (KIs) in particular are being intensively studied in clinical trials in thyroid cancer: of note, various BRAF inhibitors for tumors that bear mutation of this gene [22–24], as well as a series of multi-kinase inhibitors, are emerging as potentially effective options in the treatment of advanced TC that are not responsive to traditional therapies [10]. However, the efficacy of KIs in patients with differentiated thyroid carcinoma has given contrasting evidence in the clinical trials, probably due to the drug resistance (as in other tumor types). Furthermore, KIs might cause significant side effects. This underlines the need for further efforts aimed at the identification of novel therapeutic approaches for thyroid tumors [10].

Large-scale siRNA-based functional screening on cancer cell lines are nowadays widely used for the identification of tumor cells vulnerabilities to be explored for therapeutic purposes [12]. Interestingly, such approaches allow the discovery of target genes that are required by tumor cells in specific contexts, thus providing the premise for the identification of therapeutic approaches with no or limited effect on normal cells.

In this paper, we described, for the first time, a high-throughput siRNA phenotypic screening campaign for the identification of thyroid tumor cell vulnerabilities. By screening a “druggable genome” siRNA library, we identified 398 siRNA oligos (targeting 386 genes) which preferentially inhibited the growth of the BCPAP papillary thyroid cancer cell line as compared to that of non-transformed thyrocytes (Nthy-ori 3–1). Fifteen out of 28 hit genes were technically confirmed. The relevance of this screening study and its ability to identify bona fide cell vulnerabilities is supported by two lines of evidence. First, *BRAF* was detected among the top BCPAP vulnerability genes. This was expected, as the dependency of BCPAP cells upon *BRAFV600E* oncogene has been previously documented [16]. Second, the screen efficiently detected genes that were indiscriminately lethal for both the Nthy-ori 3–1 and BCPAP cell lines: in many cases, these were found to be genes that encode proteins of essential function and have been identified as survival genes in previous functional screenings [14, 18, 25].

Analyzing the thyroid TCGA data set, we found no mutations affecting the confirmed hit genes, besides *BRAF*, suggesting that functional alterations in PTC may be excluded. With respect to gene expression the hit genes were found to be either equally or differentially (both upregulated and downregulated) expressed in PTC in comparison with normal samples.

Three hit genes, namely *Cyclin D1*, *MASTL* and *COPZ1*, were selected for further confirmation. Their vulnerability for BCPAP cells was confirmed by using siRNA oligos different from the screening. Interestingly, we showed that the silencing of *Cyclin D1*, *MASTL* and *COPZ1* reduces the growth and survival of several different TC cell lines, regardless the histotype and the oncogenic lesion.

Cyclin D1 is an important cell cycle regulator that promotes G1/S phase transition by activation of CDK4 and CDK6 kinases [26]. Cyclin D1 is frequently overexpressed in human cancer, and it has been proposed as a therapeutic target. Several approaches aimed at its inhibition are presently ongoing. As for all cyclins, while the lack of enzymatic activity makes direct targeting of Cyclin D1 difficult, a much more amenable approach is to target cyclin functions by inhibition of their associated kinases. Indeed, several CDK4/6 inhibitors have shown promising antitumor activity in experimental systems, and are presently under clinical evaluation for different tumor types [26]. Alternative approaches are also currently under investigation, including the targeting of factors which regulate Cyclin D1 protein turnover, such as the USP2 deubiquitinase [27]. In thyroid carcinoma overexpression of *Cyclin D1* at mRNA and protein level has been documented, and has been suggested to contribute to tumor progression [28–30]. However, no functional data dissecting the role of Cyclin D1 in thyroid tumorigenesis are available. We found that *Cyclin D1* inhibition led to a consistent reduction of cell growth in all the thyroid tumor cell lines analyzed. This was not associated with Cyclin D1 expression level, suggesting that the dependency of thyroid tumor cells upon Cyclin D1 activity may not require aberrant protein expression. We also found that all the thyroid tumor cell lines are sensitive to palbociclib (PD-0332991), a selective CDK4/6 inbititor approved for the treatment of ER+/HER2- breast cancer in combination with letrozole [31]. Whether the antiproliferative effect after *Cyclin D1* silencing or palbociclib treatment in thyroid tumor cells, but not in normal ones, is related to the presence of functional pRB, as reported for other tumor types [19], remains to be investigated. Our studies identified Cyclin D1 as a therapeutic target for thyroid tumors, and provide a rationale for preclinical and clinical studies aimed to assess the efficacy of inhibitors of Cyclin D1 function in thyroid cancer.

MASTL (Microtubule associated serine/threonine kinase-like) regulates mitosis progression by inhibiting PP2A/B55δ, the principal protein phosphatase complex that dephosphorylates CDK1 substrates. MASTL activity is essential for prevention of defects in chromosome condensation and segregation, prometaphase arrest and mitotic collapse during cell division [32]. Furthermore, it modulates DNA damage response by promoting checkpoint recovery and cell cycle progression [33]. Little is known about MASTL in cancer. Nagel et al. [34] reported that the knockdown of *MASTL*, through promoting defects in in cytokinesis and a shortened G2/M phase arrest, is capable of sensitizing lung cancer cells to radiations. Notably, *MASTL* inhibition affects tumor cell but not normal primary fibroblast radiosensitivity, thus suggesting that MASTL could represent a druggable target to combine with radiotherapy. In head and neck carcinoma *MASTL* was found involved in progression and recurrence and proposed as therapeutic target [35]. We have identified *MASTL* as vulnerability gene for thyroid carcinoma. In fact, *MASTL* silencing interfered with the growth of all thyroid cell lines, although with variable extents. Moreover, evidence of failure of correct cell division process was found in MASTL-depleted cells. Of note, our study identified MASTL as a novel mitotic machinery target in thyroid tumor, in addition to PLK1 and AURKs, both overexpressed and proposed as therapeutic targets in ATC [36, 37]. Remarkably, we found that MASTL vulnerability in thyroid tumor cell lines is not associated with overexpression; on the contrary, *MASTL* is slightly downregulated in PTC samples and in thyroid tumor cells. Overall these findings highlights the need of further investigation in order to assess whether MASTL may represent a useful target in different tumor types, as well as the search/design of strategies to block the activity of MASTL, for which no specific inhibitors have been so far identified.

COPZ1 (coatomer protein complex ζ1) is a subunit of coatomer protein complex I (COPI), a secretory vesicle coat protein complex involved in Golgi apparatus and endoplasmic reticulum traffic, endosome maturation, autophagy [38, 39] and lipid homeostasis [40]. *COPZ1* has been proposed as a tumor-specific gene target. It has been shown that tumor cells become dependent on *COPZ1*, as a result of a tumor-specific downregulation of its isoform *COPZ2* [21]. Interestingly, *COPZ1* knockdown kills both proliferating and non-dividing tumor cells, but does not affect normal cells, thus suggesting that COPZ1-targeting therapies have the potential for eradicating cancer cells, independently of whether they are actively proliferating. Our screening and validation identified *COPZ1* as a thyroid tumor cell specific survival gene. Interestingly, we also found a significant downregulation of *COPZ2* mRNA expression in the majority of thyroid tumor cell lines. An exception is represented by WRO82–1 which, despite expressing *COPZ2* mRNA levels higher than control, is sensitive to *COPZ1* silencing. This raises the possibility that other mechanisms, in addition to *COPZ2* downregulation, may be involved in determining tumor cell *COPZ1* dependency. Whether the thyroid tumor cell dependency upon *COPZ1* is related to downregulation of *COPZ2* isoform, or to deregulation of other COPI components, as well as the modalities through which COPZ1 inhibition leads to growth suppression of thyroid tumor cells, remains to be investigated.

In conclusion, by a siRNA-based functional screening we have identified a signature of vulnerability for thyroid carcinoma cells, thus identifying novel potential therapeutic targets in this setting. Further characterization of three hit genes, namely *Cyclin D1*, *MASTL* and *COPZ1*, demonstrates that their abrogation selectively impairs the growth of several thyroid tumor cell lines, irrespective of the histotype or driving genetic lesion, thus indicating that they represent common examples of “non-oncogene addiction” in thyroid cancer. Variable extents of cell growth inhibition among the cell lines upon silencing of these target genes were observed. Whether these differences are related to genetic background and/or intrinsic sensitivity to cell death remains to be investigated. The dependency upon Cyclin D1, MASTL and COPZ1 activity is restricted to tumor cells and not normal thyroid cells, as previously reported for other tumor types [21, 27, 34, 35]. Thus we envisage that Cyclin D1, MASTL and COPZ1 are attractive targets for new therapeutic approaches for thyroid cancer which spare normal cells and which would thus have limited side effects.

## MATERIALS AND METHODS

### Cell lines

Nthy-ori 3–1 cell line (SV-40 immortalized normal human thyroid follicular cells) was purchased from European Collection of Cell Cultures (ECACC) (Salisbury, UK); BCPAP, TPC-1, WRO82–1, 8505C, KAT-18 were obtained from Prof. A. Fusco (University Federico II, Naples, IT); HTC/C3 was purchased from Riken Cell Bank (Tsukuba, Japan).

Nthy-ori 3–1 and the PTC-derived BCPAP cell lines were cultured in RPMI 1640 medium (Gibco Life Technologies, Carlsbad, CA, USA) supplemented with 10% (v/v) heat-inactivated fetal bovine serum (FBS) (EuroClone, Pero, Italy), penicillin (100 U/ml) and streptomycin (100 mg/ml). The other cell lines PTC-derived TPC1; FTC-derived WRO82-1; ATC-derived 8505C, KAT18 and HTC/C3 were maintained in DMEM (Gibco, Life Technologies, Carlsbad, CA, USA) medium containing 10% FBS and 2 mM glutamine. All cell lines were cultured as monolayer at 37°C in a 5% CO_2_ humidified atmosphere.

Cell lines were genotyped at the Fragment Analysis Facility of Fondazione IRCCS Istituto Nazionale dei Tumori, using Stem Elite ID System (Promega Corporation, Madison, USA) according to the manufacturer's instructions and ATCC guidelines. The profiles obtained matched to their original profiles [41, 42]. WRO82–1 profile matched to that reported by Xu et al. [43] and HTC/C3 to that reported by JCRB Cell Bank (http://cellbank.nibio.go.jp/legacy/celldata/jcrb0164.htm).

Mycoplasma contamination was tested periodically and found negative in all cell lines (PCR Mycoplasma Detection Set, TAKARA Bio Inc).

### siRNA transfection optimization

Optimal conditions for reverse siRNA transfection of Nthy-ori 3–1 and BCPAP cells were determined by comparing cell viability after transfecting a positive control siRNA targeting the proteasome subunit PSMC3, and a negative control non-targeting oligo (siNT), in the presence of different cell densities and Lipofectamine RNAiMAX reagent (Invitrogen Life Technologies Carlsbad, CA, USA) concentrations. The best reagent and transfection conditions were those that produced the least (<10%) cell loss with siNT, and the greatest (>90%) cells decrease with the lethal PMSC3 oligo.

### High throughput siRNA screening

The human Silencer Select Druggable Genome siRNA Library V4 (Ambion Life Technologies, Carlsbad, CA, USA) is composed by 309 96-well plates containing 25139 unique lyophilized siRNA oligos targeting 9031 human genes (three oligos per gene, on average). Stock plates were obtained by dissolving oligonucleotides with 100 μl of DEPC-treated water (Ambion Life Technologies, Carlsbad, CA, USA) (final concentration 2.5 μM) and by adding NT and PSMC3 siRNA oligos (2.5 μM) in alternate position in the empty column 12 of each plate (see Figure 1A). Library oligonucleotides were further diluted to 800 nM with DEPC-treated water (mother plates). Daughter plates were prepared by transferring 17 μl/well of mother plate oligonucleotide solution in plates prefilled with 119 μl of Opti-MEM^®^ I medium (Gibco Life Technologies, Carlsbad, CA, USA) containing 4 μl/ml of lipofectamine^®^ RNAiMAX. The solution was gently mixed by pipetting and incubated for 20 minutes room temperature, and then 30 μl/well were split into four transfection plates, to which 120 μl/well of cell suspension (1250 cells /ml) were added. Two plates for each cell line (Nthy-ori 3–1 and BCPAP) were used. Each well contained 150 cells, 20 nM oligo, RNAiMAX 0.1% vol/vol. After transfection, plates were kept at room temperature for 15–20 minutes to minimize uneven distribution of the cells in each well [44], and then incubated at 37°C for 7–8 days by stacking in alternate orientations to limit uneven medium evaporation and to facilitate gas exchange. As a result of this procedure, no edge effects or evidences of asymmetric cell growth emerged from screening data analysis, and visual inspection of plate scans confirmed homogeneous cell distribution in all plates. The following custom-barcoded 96-well microplates purchased from Greiner Bio-One (Frickenhausen, Germany) were used: U-bottom microplate for mother and daughter plates; CELLSTAR^®^, Black /μClear^®^ plate for transfection plates. All liquid handling and plate tracking were performed with a Freedom EVO^®^ robotic platform (Tecan, Männedorf, Switzerland); disposable MCA384 Tips and reservoirs were purchased from Tecan.

### siRNAs

Information on siRNAs used in the transfection setup, as control in the screening, or in confirmation and validation studies is summarized in the Table S4. Oligos produced in house were designed and synthesized with stabilizing modifications (i.e.: phosphorylation in 5′ position of the antisense strand and 2′-*O*-methyl ribosyl substitution at position 2 of the sense strand) known to reduce off-target effect [45].

### Colony formation assay (CFA)

Seven-eight days after transfection, cells were fixed with formaldehyde 3.7% v/v solution at room temperature for 30 minutes, washed with PBS and stained for 20 minutes with a PBS solution containing 0.05% v/v Triton^®^ X-100 (Sigma-Aldrich, St. Louis, MO, USA) and TO-PRO^®^-3 iodide (Invitrogen Life Technologies (Carlsbad, CA, USA) diluted 1:1000. After a second wash, 50 μl PBS were left in each well. Plates were sealed and analyzed with an Odyssey^®^ infrared scanner (LI-COR Biosciences, Lincoln, NE, USA) which provides whole-well fluorescence quantification of cells stained with TO-PRO^®^-3 dye. The fluorescence intensity (FI) was considered as readout, which is proportional to both colony number and size.

### Screening data analysis

FI values were registered in a proprietary Oracle database through Symyx Assay Explorer^®^ and matched with the library information. The FI of each sample was divided by the average FI of the three non-targeting oligos within the same plate (set as 100%) for interplate normalization. TIBCO SpotFire (Boston, MA, USA) and Microsoft Excel (Redmond, WA, USA) were used for statistical analysis and visualization of screening data distributions. The means and standard deviations of FI (% siNT) values and z*-scores [46] were calculated from the two technical replicates for each cell line and reported in Table S1 (Excel file with screening results).

To identify genes whose RNAi-mediated inhibition affects the growth of BCPAP but not Nthy-ori 3–1 cells, a distance factor (*d*) expressed as:

$$d=\sqrt{{\left(100-\%\;{\text{NTO}}_{Nthy-ori\;3-1}\right)}^{2}+{\left(\%\;{\text{NTO}}_{BCPAP}\right)}^{2}}$$

was introduced. “*d*” represents the distance between the %NT value of any given siRNA oligo and the point of maximal lethality towards BCPAP without effects on Nthy-ori 3–1 (coordinate %NT_Nthy-ori 3–1_ = 100, %NT_BCPAP_ = 0). Screening hits were selected among siRNA oligos with significantly low *d* values, whereas oligos either equally inactive on both cell lines, or equally lethal on both cell lines, or preferentially lethal on Nthy-ori 3–1, are excluded. Based on the distribution analysis of *d* scores, a −3σ threshold (corresponding to *d* = 47.2) in the primary screening, and a −2σ threshold (corresponding to *d* = 49.8) in the confirmation study, were applied to define hits.

### Cell proliferation assay

Cells (500 cells/well) were reverse transfected in 96-well plate with 20 nM specific siRNAs using the Lipofectamine RNAiMAX reagent, according to manufacturer's instruction. Ten days later, cell viability was assessed by CellTiter-Glo Luminescent Cell Viability Assay (Promega Corporation, Madison, USA), performed as recommended by the supplier. Luminescence signals were acquired using a microplate reader (TecanUltra, Tecan Trading AG, Switzerland).

For the trypan blue exclusion assay, BCPAP and 8505C (1 × 10^5^), TPC-1 (6 × 10^4^), WRO82–1, HTC/C3 and KAT-18 (8 × 10^4)^ cells were transfected the day later in 6-well plate with 20 nM of specific siRNAs using Lipofectamine RNAiMAX, according to manifacturer's instruction. At different days after transfection (4 days for TPC1, 8505C and HTC/C3 cells; 5 days for KAT-18 cells, 6 days for BCPAP cells, 7 days for WRO82–1 cells) the fraction of viable and dead cells was analyzed. For palbociclib experiments, cells (8000 cells/well) were seeded in black/clear bottom 96-well plates and, the following day, treated with increasing doses of the palbociclib (PD 0332991) inhibitor (Selleck Chemicals, Houston, TX, USA, cat. S2768). After 72 hours, cells were fixed with 3.7% paraformaldehyde solution for 30 minutes at RT, washed and stained with 1 mg/ml Hoechst 33342. The plates were analyzed with an ArrayScan VTI high-content screening reader (Thermo-Fisher Scientific, Pittsburgh, PA): at least 800 cells were acquired in each well with a 10X objective in one fluorescence channel (XF93 optical filter set). Cell nuclei were automatically recognized and counted in ten fields to estimate the cell number per well as proliferation readout. IC50 values were calculated by sigmoid curve fitting of experimental points.

### Gene Ontology and gene expression analyses

The functional annotation analysis on the identified gene lists was performed by means of the Database for Annotation, Visualization and Integrated Discovery (DAVID) tool v6.7 (https://david.ncifcrf.gov/). Gene Ontology (GO) Biological Process (BP) and Molecular Function (MF) terms were used as annotation categories and medium classification stringency was set for the functional annotation clustering analysis. The annotation clusters with an Enrichment score (ES) >1.3 were selected and representative GO-terms were indicated for each significant cluster. To assess the interactions between the validated fifteen genes, network analysis was carried out using the Ingenuity Pathway Analysis Tool (Ingenuity^®^ Systems, http://www.ingenuity.com). The Core Analysis function was used to compare genes pooled from literature and other molecules in IPA's database.

The thyroid TCGA data set, containing the molecular data on nearly 500 PTC, is the largest data collection so far available (https://tcgadata.nci.nih.gov/tcga/tcgaCancerDetails.jsp?diseaseType=THCA&diseaseName=Thyroid). Illumina HiSeq Level 3 data were downloaded from TCGA Data Portal and RSEM [47] gene normalized expression values of selected genes were derived for 58 PTC patients, in tumor and adjacent normal samples, respectively. The statistical significance of gene expression difference between PTC and normal groups was assessed using Wilcoxon test.

### RNA extraction, RT-PCR and Real time PCR

RNA extraction and RT-PCR were performed as previously described [48], using the following primer: 5′-ATGAGAGGCCTGTGGAGCACT-3′ (forward), 5′-TCATGTGTCGACTTGCAGGAG-3′(reverse) for RGS3 fragment amplification; 5′-GCAGATCCTCAGATCAGTGAA-3′(forward), 5′-TATAATGGGATCTGCAGCGTG-3′ (reverse) for NUDT9; 5′-CGAGGTCTACCAGCTCGACAT-3′ (forward), reverse 5′-CACGTCCACGTTCTCCTTGGT-3′ (reverse) for RASD1; 5′-ATGTGGAGCGTCTGGGATGC-3′ (forward), 5′-GCTCAGGACAAAGGAGGACG-3′ (reverse) for OXTR [49].

For Real Time PCR analysis 20 ng of retrotranscribed RNA were amplified in PCR reactions carried out in triplicate on an ABI PRISM 7900 using TaqMan gene expression assays (Applied Biosystem, Foster City, CA). Hs00212698_m1 was used for COPZ2 expression; human HPRT1 (HPRT-Hs99999909_A1) was used as housekeeping gene for the normalization among samples. Data analysis was performed using the SDS (Sequence Detection System) 2.4 software.

### Western blot analysis

Western blot analysis was performed as previously described [48], using the following antibodies: anti-Cyclin D1 (DCS-6: sc-20044, 1:500), anti-COPE, (E-20: sc-12104, 1:500), anti-PLA2G15 (LYPLA3, H-167: sc-135297, 1:500), anti-MAP4K5 (KHS, N-19: sc-6429, 1:1000), anti-COPZ1 (D-20: sc-13349, 1:750), anti-GAPDH (6C5, sc-3223: 1:2000) purchased from Santa Cruz, CA, USA; anti-MASTL (ab86387, 1:7000) from Abcam, Cambridge, UK; anti-β-actin (A2066, 1:5000) and anti-vinculin (V9131, 1:1000) from Sigma-Aldrich, St Louis, Mo, USA.

### Immunofluorescence analysis

Cells growing on glass coverslips were transfected with 20 nM NT or MASTL siRNAs and, 48 hours later, fixed for 10 minutes with 4% paraformaldehyde (Sigma Aldrich, St Louis, Mo, USA). After permeabilization for 10 minutes with 1% BSA and 0.1% Triton X-100 in PBS, and incubation for 30 minutes with 1X blocking solution (2% BSA in PBS), cells were incubated with anti-β-tubulin (clone TUB 2.1: T4026, 1:400, Sigma Aldrich, St. Louis, MO, USA) or anti-histone H3 (phospho S10) (E173: ab32107, 1:1000, Abcam, Cambridge, UK) primary antibodies for 1 hour. After washing with PBS, cells were incubated with Alexa Fluor^®^ 546 rabbit (1:500, Invitrogen/Molecular Probes^®^) and Alexa Fluor 488^®^ mouse (1:500, Invitrogen/Molecular Probes^®^) secondary antibodies for 1 hour. Slides were then prepared using ProLong Diamond Antifade mountant with DAPI (P36966, Molecular Probes^®^) and imaged with immunofluorescence microscopy (Eclipse E1000; Nikon Instruments, Inc. NY, USA).

## SUPPLEMENTARY FIGURES AND TABLES








