# Supplementary material for: Identification of thyroid tumor cell vulnerabilities through a siRNA-based functional screening
Source: Oncotarget. 2015 Sep 25;6(33):34629–48. doi: 10.18632/oncotarget.5282 (PMC4741478; doi:10.18632/oncotarget.5282)
Supplement: Supplementary file 1 [file oncotarget-06-34629-s001.pdf]

SUPPLEMENTARY FIGURES AND TABLES

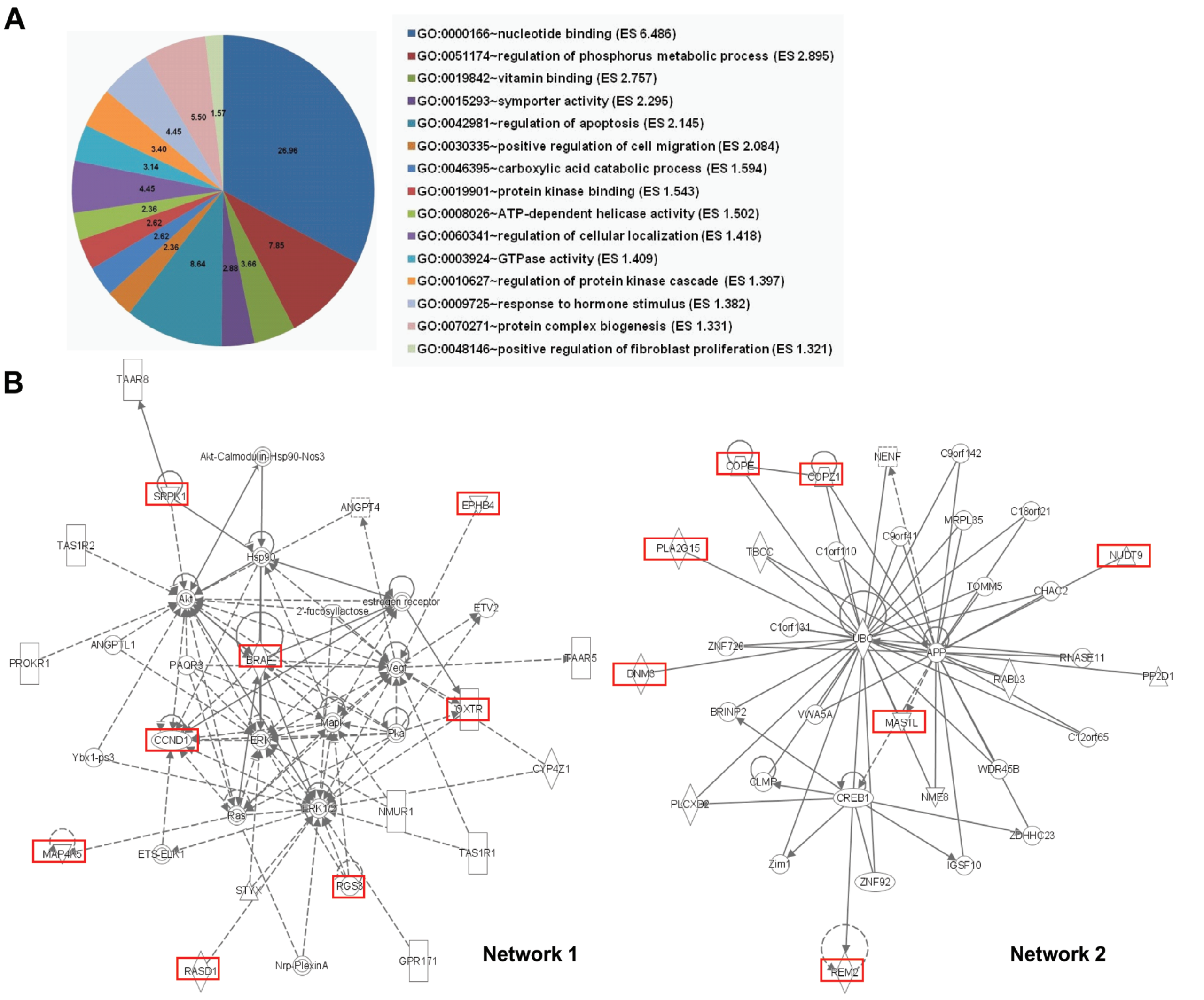

Supplementary Figure S1: A. Pie chart of functional annotation clustering analysis on 386 genes by DAVID 6.7 tool. Only 15 annotation clusters with an Enrichment Score (ES) > 1.3 are reported. A representative Biological Process (BP) or Molecular Function (MF) GO-term and the percentage of the genes on the 382 DAVID IDs globally identified are indicated for each significant annotation cluster; for each selected cluster the ES is also indicated. **B.** Visual representation of the two principal networks generated by Ingenuity Pathway Analysis (IPA) including the fifteen validated hits. Solid lines represent a direct interaction between the two gene products and dotted lines mean an indirect interaction.

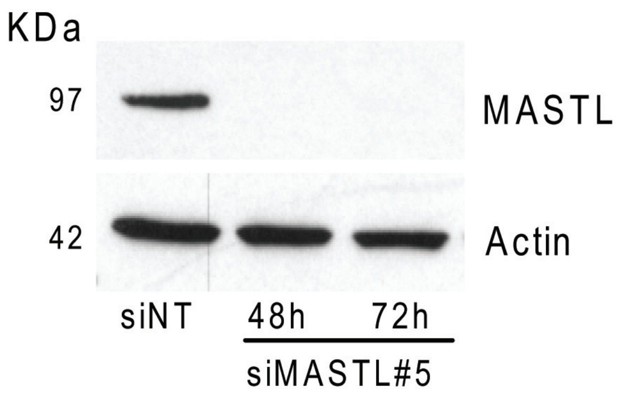

Supplementary Figure S2: western blot analysis of MASTL protein expression in Nthy-ori 3–1 cells, transiently transfected with MASTL#5 siRNA; actin was used as loading control.

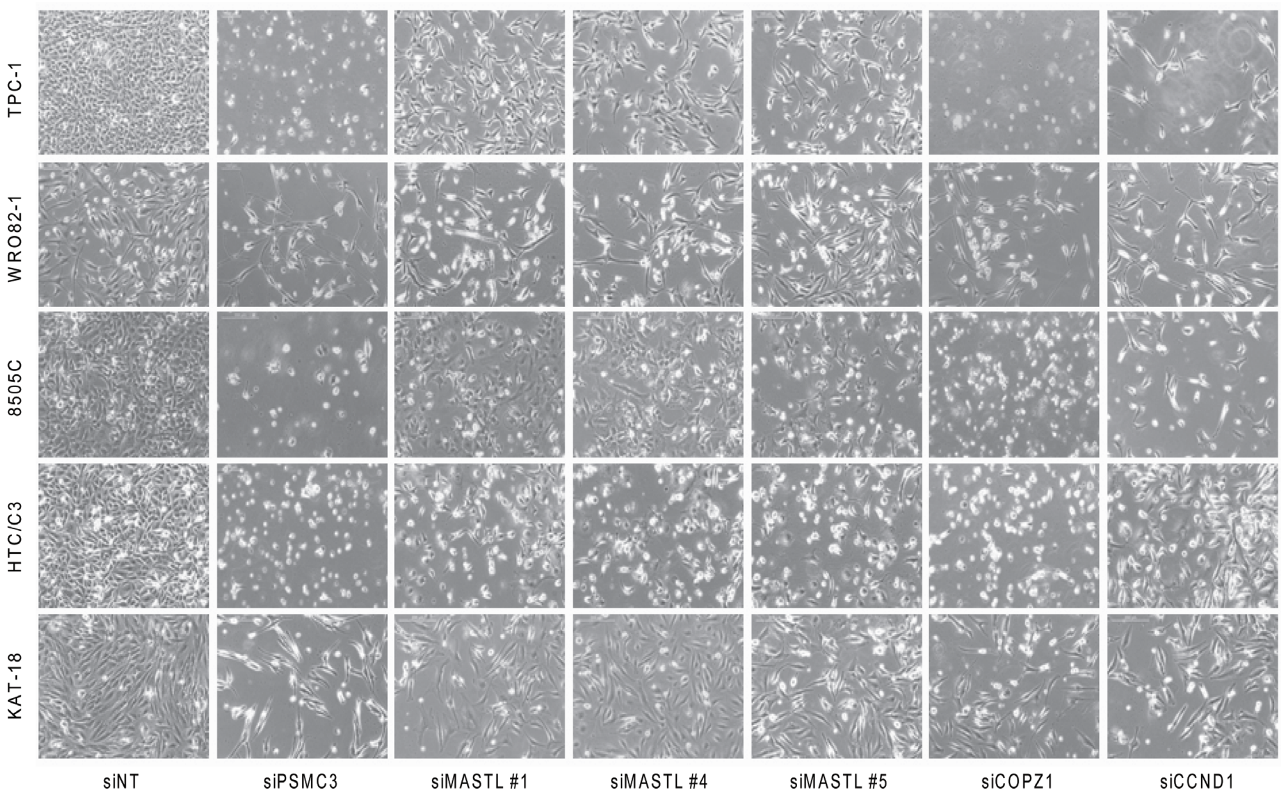

Supplementary Figure S3: representative pictures at 10X magnification of TPC-1, WRO82–1, 8505C, HTC/C3 and KAT-18 cells, taken before the trypan blue exclusion assay

Supplementary Table S1: List of 25139 siRNA oligonucleotides of the human Silencer Select Druggable Genome siRNA Library V4 (Ambion Life Technologies, Carlsbad, CA, USA) and screening results. Average and standard deviation (StDev) of the fluorescence intensity (FI) normalized on siNT (%NT), and average and standard deviation (StDev) of z* scores for BCPAP and Nthy-ori 3–1, distance factor (*d*) and binned *d* (based on σ distribution) are shown. The gene symbols, full gene names, Refseq (http://www.ncbi.nlm.nih.gov/refseq/about/) accession numbers, siRNA batches and their corresponding plate blocks were provided by Ambion.

Supplementary Table S2: List of 398 siRNA oligonucleotides resulted to preferentially inhibit the proliferation of BCPAP cells with respect to Nthy-ori 3–1 (d < -3σ) and thus defined as "differential hits".

Supplementary Table S3: List of 1695 siRNA oligonucleotides resulted to inhibit the proliferation of both BCPAP and Nthy-ori 3–1 (signal intensity < 20% siNT), therefore defined as “lethal hits”.

Supplementary Table S4: siRNA oligos.

siRNA	Sequence/catalog number	Source	Used in
**siNT**	5′-UGGUUUACAUGUCGACUAAtt-3′	in house	transfection setup, screening, confirmation
**siPSMC3**	5′-CGGCUGAAGUGCGCAAUAAtt-3′	in house	transfection setup screening, confirmation
**siNT**	ON-TARGET plus Non-targeting Pool siRNA D-0018–10–05	ThermoScientific, Dharmacon Inc., Chicago, IL, USA	validation
**siPSMC3**	MISSION esiRNA EHU107331	Sigma-Aldrich, St. Louis, MO, USA	validation
**siMASTL #1**	ON-TARGET plus set of 4 siRNAs J-004020–12	ThermoScientific, Dharmacon Inc., Chicago, IL, USA	validation
**siMASTL #2**	ON-TARGET plus set of 4 siRNAs J-004020–11	ThermoScientific, Dharmacon Inc., Chicago, IL, USA	validation
**siMASTL #3**	ON-TARGET plus set of 4 siRNAs J-004020–10	ThermoScientific, Dharmacon Inc., Chicago, IL, USA	validation
**siMASTL #4**	ON-TARGET plus set of 4 siRNAs J-004020–09	ThermoScientific, Dharmacon Inc., Chicago, IL, USA	validation
**siMASTL#5**	5′-UGUGGAUUCUGGUGGGAUAdTdT 3′	in house	validation
**siCCND1**	MISSION esiRNA EHU153321	Sigma-Aldrich, St. Louis, MO, USA	validation
**siCOPZ1**	MISSION esiRNA EHU1040461	Sigma-Aldrich, St. Louis, MO, USA	validation

List of siRNA oligos used in the transfection setup, as control in the screening, or in confirmation and validation studies
